# Cost-Effectiveness Analysis of Transanal Irrigation for Managing Neurogenic Bowel Dysfunction in Japan

**DOI:** 10.36469/9781

**Published:** 2018-02-12

**Authors:** Atsushi Sengoku, Shinichi Noto, Masashi Nomi, Anton Emmanuel, Tatsunori Murata, Toshiki Mimura

**Affiliations:** 1 Department of Urology, Hyogo Rehabilitation Center Hospital, Japan; 2 Department of Occupational Therapy, Faculty of Medical Technology, Niigata University of Health and Welfare, Japan; 3 GI Physiology Unit, University College London Hospital, London, United Kingdom; 4 CRECON Medical Assessment Inc., Tokyo, Japan; 5 Center for Functional Bowel and Anorectal Disorders, Sashiogi Hospital, Japan

**Keywords:** Spinal Cord Injury, Neurogenic bowel dysfunction, Transanal irrigation, Peristeen, Quality adjusted life years, Cost-effectiveness, Japan

## Abstract

**Background:**

Neurogenic bowel dysfunction (NBD) is a common sequela in Spinal Cord Injury (SCI) patients. Bowel dysfunction symptoms have a significant negative impact on quality of life (QOL) and are often socially disabling. Transanal irrigation (TAI) is a bowel management procedure that significantly mitigates NBD symptoms in patients refractory to standard bowel care (SBC) by reducing the incidence of fecal incontinence, ameliorating constipation, and improving QOL. TAI devices are used across many countries such as the United Kingdom, Germany, and France, and introduction of the devices is being considered in Japan. In this context, a cost-effectiveness analysis specific to Japanese settings is relevant.

**Objectives:**

To analyze the cost-effectiveness of TAI for bowel management of SCI patients with NBD in a Japanese clinical setting.

**Methods:**

A modified version of a previously developed and published Markov model was used to evaluate the cost-effectiveness of TAI. In the model, SCI patients using TAI due to NBD were compared with SCI patients not responding to TAI and continuing with SBC. Quality-adjusted Life Years (QALYs) were used as the primary effectiveness measure, and the analysis was conducted from the payer’s perspective.

**Results:**

The model predicts a lifetime incremental cost of TAI to be 3 198 687 yen compared with SBC. TAI provided an additional 0.8 QALY, which leads to an incremental cost-effectiveness ratio (ICER) of TAI vs SBC of 4 016 287 yen/QALY.

**Conclusions:**

An ICER of 4 million yen falls within the range of reported willingness to pay (WTP) per QALY gain (5–6.7 million yen) in Japan, and TAI is therefore found to be a cost-effective treatment strategy compared to SBC. The result should be further corroborated in future Japanese trials of TAI.

## Introduction

Nerve injury, neurological disease, or congenital defects of the nervous system that causes loss of normal bowel function is termed neurogenic bowel dysfunction (NBD). It usually includes combinations of fecal incontinence, constipation, abdominal pain, and bloating.^1^,^2^ NBD greatly impacts patients’ quality of life (QOL) as well as their social lives.^3^,^4^,^5^ Several studies across the world have indicated that appropriate bowel management can significantly improve NBD.^6^–^13^ A stepped approach to bowel management has been recommended, consisting of “conservative” management options (eg, diet and lifestyle alterations), “minimally invasive” options (TAI) and “more invasive options” (eg, sacral nerve stimulation [SNS] or stoma creation).^14^ The stepped approach is intended to offer patients improved NBD through the least invasive method.

Transanal irrigation (TAI) is a bowel management procedure that involves transanal installation of water into the rectum/colon inducing bowel contractions with subsequent removal of feces in the rectum and left side colon.^15^ Regular removal of feces using TAI is intended to prevent fecal incontinence and constipation and has been shown to reduce urinary tract infections (UTIs) and the risk of stoma surgery.^16^,^17^ Patients can perform TAI by themselves promoting autonomy by allowing them to choose a place and time for defecation. Altogether, TAI improves the QOL and reduces the time spent on bowel management compared with conventional standard bowel care (SBC).^18^,^19^ TAI has also been recommended in international guidelines as a safe method for improving constipation and fecal incontinence in individuals with neurogenic bowel dysfunction.^13^

TAI is increasingly used as a minimally invasive treatment strategy for NBD and specially developed integrated systems with a rectal balloon catheter and a water pump/container allow patients with reduced hand function and/or reduced mobility to irrigate themselves. Introduction of TAI devices in Japan as a new bowel management regime is believed to improve the QOL of Japanese patients with NBD as well as to ameliorate their bowel symptoms.

However, the introduction of TAI to the Japanese market involves additional expenses in terms of cost for the devices as well as costs for patient training and follow-up. As health practice and costs vary between countries, it is imperative to obtain local Japanese data to evaluate device-assisted TAI as a potential bowel management strategy and provide a basis for adequate reimbursement. Therefore, a cost-effectiveness analysis was conducted in Japanese SCI patients with NBD.

## Method

### Design of Decision Model

**Figure 1:**
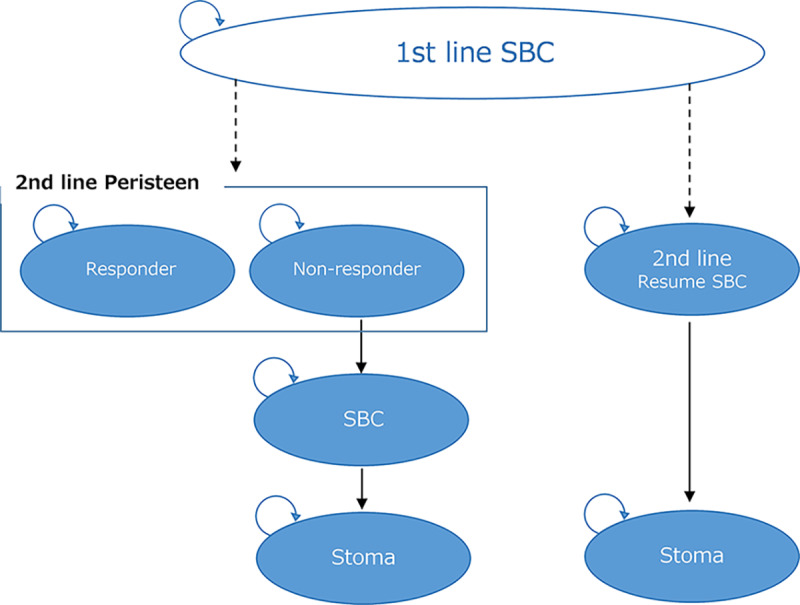
Markov model SBC: standard bowel care

A Markov model, previously developed and published in the United Kingdom,^17^ was used in this analysis. The model was further modified to suit the Japanese clinical settings and was used to evaluate the cost-effectiveness of SCI patients using TAI in comparison with SBC as a treatment for NBD (Figure 1). In the model of the previous study,^17^ surgical interventions such as SNS, sacral anterior root stimulation (SARS), and antegrade continence enema (ACE) were considered. However, in this study, these surgical interventions were excluded from the model because they are rarely used in Japan for NBD patients and are thought to be inappropriate for inclusion in the model.^20^

In the analysis, TAI patients are defined as patients who use TAI as a second line treatment after first-line treatment of SBC has failed. In the model, TAI-patients were categorized in two patient groups: patients who experience good bowel management (responders) and insufficient bowel management (non-responders). The responders were assumed to experience satisfactory bowel function throughout the time horizon and did not undergo stoma surgery. Non-responders were assumed to resume SBC after termination of TAI as a treatment and therefore be in risk of undergoing stoma surgery. In the SBC group, patients who were refractory to SBC were assumed to undergo stoma surgery.

A cycle length of 6 months was applied to the model throughout a lifetime time horizon, corresponding to the length of time a patient had to spend on failing SBC to be assessed as eligible for TAI.^17^ Following Japanese guidelines for cost effectiveness analysis, the analysis was conducted from the health care payer’s perspective.^21^ Quality-adjusted Life Years (QALYs) were used as an effectiveness measure, and only direct medical costs incurred for the treatment were considered in the analysis. Annual discount rates of 2% were included in the model for incurred costs and treatment effectiveness.^21^

### Parameters

In the given model, the Neurogenic Bowel Dysfunction score (NBDS) for each individual health state was applied to determine the utility weight. NBDS is a symptom-based score for NBD developed among spinal cord injury (SCI) patients and covers both constipation and fecal incontinence.^22^ Each parameter (such as clinical parameters, utility weights, and cost parameters) was included based on published data in literature and information from government agencies. When relevant information on clinical parameters or utility weights was unavailable, the parameters were instead calculated based on a survey for SCI patients suffering from bowel dysfunction. The web-based survey was conducted in co-operation with the Non-profit Organization Japan Spinal Cord Foundation, the Association Spinal Injuries Japan, and the Spina Bifida Association of Japan. The survey included 275 respondents and the method as well as main results were previously reported.^20^ The survey aimed at obtaining information on the current economic and health status of the patients, their bowel and urinary function, labor productivity, and disability. Bowel dysfunction in patients was evaluated based on the NBDS, labor productivity and disability were evaluated using Work Productivity and Activity Impairment Questionnaire: Specific Health Problem (WPAI:SHP), and utility was evaluated using the 5-level EQ-5D version (EQ-5D-5L) and the Health Utility Index Mark 2/3 (HUI3). To ensure data from the TAI eligible patients, only the 217 patients, out of the total 275 respondents, meeting the inclusion criteria of Christensen *et al*., were included in the current analysis.^16^ The criteria were 1) aged >18 years, 2) at least 3 months since onset of spinal cord injury, 3) patients with one of the following conditions: defecation time of >30 minutes; fecal incontinence once or more per month; symptoms reflecting autonomic dysreflexia or abdominal discomfort before or during defecation; and patients with an NBDS of >6. Patients who failed to answer all questions or provided different answers to similar questions were also excluded. Characteristics of the patients included in the analysis are shown in Table 1.

Where relevant information on cost parameters was unavailable, it was calculated based on the responses to a cost and treatment practice questionnaire filled out by four Japanese medical professionals specializing in SCI or bowel management. These specialists are the authors (AS, SN, MN, TM) of this study. The aim of the cost and treatment practice questionnaire was to obtain information on the treatment for NBD in Japan as well as treatment in case of UTIs and stoma surgery. Each treatment cost was calculated using the Japanese medical fee schedule or drug prices and based on the responses to the questionnaire.

#### 1) Clinical Parameters

The clinical parameters determined in this study are listed in Table 2.

##### TAI-mediated Improvement of NBDS

Improvement in NBDS was considered as the main outcome of TAI-usage in the current model. A randomized controlled trial (RCT) in patients with SCI has compared the effectiveness of TAI performed with the Peristeen Anal Irrigation system (Coloplast A/S, Kokkedal, Denmark) with SBC.^16^ The results showed a significant overall improvement (3 points) of NBDS in the Peristeen-TAI group compared to the SBC group. However, data from TAI-responders or non-responders were not reported in this study. To account for this lacking information, an additional NBDS-analysis was carried out on the data-set used in the original cost effectiveness analysis from the UK published by Emmanuel *et al*.^17^ This prospective “real world data-set” was collected in three clinics in the UK between 2007–2014 and includes 227 patients using Peristeen-TAI due to bowel dysfunction from either SCI, cauda equina syndrome, multiple sclerosis, or spina bifida. Peristeen-TAI was found to be suitable for the 227 patients if they had previously failed SBC for at least six months. Since the current analysis focuses on SCI, the additional NBDS-analysis was carried out on the 130 patients in the SCI-subpopulation. This group consisted of 77 men and 53 women with an average age of 43 years (range: 17–82 years) and 8.5 years (range: 0,5–20 years) since being diagnosed. The analysis shows that NBDS improved by approximately 6 points in case of Peristeen-TAI responders and deteriorated by approximately 1 point in case of non-responders in comparison with their baseline before starting Peristeen-TAI.

The baseline NBDS of the SBC group was obtained from the responses to a web-based questionnaire.

The proportion of responders and non-responders in the TAI group over time has previously been reported in a long-term study, and the withdrawal rate of patients from TAI use per cycle (non-responders) was calculated based on the rate of patients (responders) who continued on TAI in this study.^18^

##### Reduction in the Risk of UTI When Using Peristeen-TAI

The survey questionnaire helped to determine the prevalence of UTIs, rate of hospitalization among patients with UTI, number of outpatients, and proportion of untreated outpatients. The effect of UTI risk reduction by using TAI was applied to the UTI prevalence reported by patients in the survey as a relative risk calculated from the UTI prevalence of TAI and SBC respectively reported in a previous study.^16^

##### Reduced Hospitalization Due to Peristeen-TAI Usage

Hospitalization due to pressure ulcer in NBD patients is considered to be lengthy and expensive. Therefore, hospitalization due to pressure ulcer was separated from hospitalizations due to other reasons in the model. Hospitalization frequency per cycle due to pressure ulcer and other reasons were determined from the results of the survey. The TAI-mediated relative risk was calculated from the data reported by Emmanuel *et al*. on the frequency of hospitalization among TAI and SBC users.^17^

##### Reduction in the Nursing Visit Rate Due to Improved NBDS

Analysis of the survey revealed a statistically significant association between the reduction in the nursing visit rate and improvement in NBDS (p=0.0006). Therefore, the effect of TAI use on the reduction in the rate of nursing visits was factored into the model using a regression equation developed by logistic regression analysis. This equation considered TAI-mediated improvement in NBDS and nursing visit rate, while age, gender, and function of hands were included as additional covariates.

##### Incidence of Stoma

The survey was used to create a Kaplan-Meier curve for stoma events. The curve was created based on the following information: “duration of disability (SCI, spina bifida, etc.) resulting in NBD” (duration until the endpoint: patients who did not undergo stoma surgery), and “duration of suffering from bowel dysfunction that led to stoma construction” (duration until the event occurrence: include patients who underwent stoma surgery). This information was also used to calculate the exponential rate of stoma incidence per cycle in the model. Since those patients who experienced unsuccessful bowel management with SBC undergo stoma surgery, it was assumed that the stoma incidence rates at the resume SBC state in the Peristeen-TAI group and SBC group are equal.

#### 2) Utility Weight

A literature search showed that no published data on the impact of TAI on health-related QOL among Japanese patients with SCI exist. The influence of bowel dysfunction on the overall health (utility) of SCI patients was therefore examined in a mapping algorithm between the NBDS-data and EQ-5D-5L-data from the survey among SCI patients (Table 3). Using a Monte Carlo simulation, the mapping was conducted based on the selection probability estimates at each individual level and in each dimension of EQ-5D-5L which were calculated from the multinomial logistic regression analysis for NBDS.

Age and gender were used as covariates in the multinomial logistic regression analysis. The Monte Carlo simulation was conducted based on the method used by Rivero-Arias *et al*,^23^ while the expected utility weight at each NBDS was calculated by repeating virtual experiments on which level to choose in each attribute of EQ-5D-5L. This was done in accordance with the conditional expression below based on random numbers (ui) which return a value in extent of zero to one.

$$\begin{array}{l} Predicted\;value\;of\;level\;in\;EQ-5D-5L\;({\hat{y}}_{i})=\\ \{\begin{array}{c} 1\;if\;u_{i}\leq P({\hat{y}}_{i}=1|NBDS_{j})\\ 2\;if\;u_{i}>P\left({\hat{y}}_{i}=1|NBDS_{j}\right)\;and\;u_{i}\leq \sum_{k=1}^{2}P\left({\hat{y}}_{i}=k|NBDS_{j}\right)\\ 3\;if\;u_{i}>\sum_{k=1}^{2}P\left({\hat{y}}_{i}=k|NBDS_{j}\right)\;and\;u_{i}\leq \sum_{k=1}^{3}P\left({\hat{y}}_{i}=k|NBDS_{j}\right)\\ 4\;if\;u_{i}>\sum_{k=1}^{3}P\left({\hat{y}}_{i}=k|NBDS_{j}\right)\;and\;u_{i}\leq 1-P\left({\hat{y}}_{i}=5|NBDS_{j}\right)\\ 5\;if\;u_{i}>1-P\left({\hat{y}}_{i}=5|NBDS_{j}\right) \end{array} \end{array}$$

SAS 9.4 was used to conduct the multinomial logistic regression analysis, while Microsoft Excel was used for the Monte Carlo simulation. The number of Monte Carlo simulation trials was 10 000 for each individual level of NBDS (NBDSj = 0–47).

#### 3) Cost Parameters

Since the majority of the clinical evidence on TAI, including the RCT by Christensen *et al*.^16^ and the original cost-effectiveness analysis by Emmanuel *et al*.,^17^ is based on the Peristeen Anal Irrigation system, this was also used for cost calculation. The Peristeen Anal Irrigation system has not yet been introduced in Japan, and the device costs were hence calculated in accordance with the average market prices in the United Kingdom, Germany, and France. The cost parameters are shown in Table 4.

No instruction/procedure fee exists for TAI today, and it was therefore assumed in the base case analysis that a new “self-defecation care instruction fee” would be equal to the existing procedure fee in Japan “C106 home self-catheterization care instruction fee (18 000 yen)” and the “C110-4 home sacral nerve stimulation care instruction fee (8100 yen).” These two fees were used for sensitivity analysis together with the average of both instruction fees which is 13 050 yen.

The costs incurred during the second line of treatment (UTI treatment, stoma surgery, or stoma management) in non-responding NBD patients who resumed SBC were gathered from the cost and treatment practice questionnaire filled out by the clinical experts among the authors.

Costs related to hospitalizations due to pressure ulcer or any other reasons were obtained from the data provided by Medical Data Vision Inc. (MDV). The characteristics of the patients included in the MDV data analysis are reported in Table 5.

### Analysis

To investigate the societal impact of TAI and NBD, this study also includes a scenario analysis of productivity loss due to bowel problems. Information on the rate of employment in NBD patients, the rate of productivity loss due to health-related absence from work (absenteeism), and the rate of productivity loss while being present at work due to health problems (presenteeism) were obtained from the patient survey. The wage per month was adjusted with the proportion of average wage per month for both disabled and healthy workers as mentioned in the Annual Report on Government Measures for Persons with Disabilities and the wage reported in Basic Survey on Wage Structure (Table 6).

A one-way sensitivity analysis with a variation range of ±20% of the base case settings was conducted to determine the impact on the results of each parameter. In addition, probabilistic sensitivity analysis using 10 000 Monte Carlo simulations was conducted to evaluate the uncertainty of the results. For the probability distribution of each parameter, the cost data was assumed to have a gamma distribution, while the utility weights and the event rates were assumed to follow the beta distribution.

## Results

### 1) Base Case Analysis

The results of the base case analysis are shown in Table 7. The expected QALYs gained per NBD patient in a lifetime perspective was found to be 11.80 in the TAI group and 11.00 in the SBC group, which indicates an incremental effect of 0.80 QALY with Peristeen-TAI usage.

The total cost incurred over a lifetime by each patient at a TAI procedure fee of 8100 yen, 13 050 yen, and 18 000 yen per month was 14 020 407 yen, 14 875 603 yen, and 15 730 798 yen, respectively, in the TAI group as compared with 12 532 111 yen in the SBC group, which suggests an extra cost of 1 488 297 yen, 2 343 492 yen, and 3 198 687 yen, respectively, with TAI usage. The incremental cost-effectiveness ratio (ICER) of the TAI group when compared with that of the SBC group was 1 868 712 yen/QALY, 2 942 499 yen/QALY, and 4 016 287 yen/QALY for TAI procedure fees set at 8100 yen, 13 050 yen, and 18 000 yen, respectively.

### 2) Scenario Analysis

The results of the societal perspective scenario analysis are shown in Table 8. The productivity loss (absenteeism) and the overall productivity loss (absenteeism and presenteeism) are shown both separately and in combination.

For absenteeism, the total cost incurred by each patient at a TAI procedure fee of 8100 yen, 13 050 yen, and 18 000 yen per month was 15 763 993 yen, 16 619 188 yen, and 17 474 384 yen, respectively, in the TAI group as compared with 15 482 680 yen in the SBC group, which suggests an extra cost of 281 314 yen, 1 136 508 yen, and 1 991 704 yen, respectively, with TAI usage. The ICER of the TAI group when compared with that of the SBC group was 353 219 yen/QALY, 2 942 499 yen/QALY, and 4 016 287 yen/QALY when the TAI procedure fee is set at 8100 yen, 13 050 yen, and 18 000 yen, respectively.

For combined absenteeism and presenteeism, the total cost incurred by each patient at a TAI procedure fee of 8100 yen, 13 050 yen, and 18 000 yen per month was 31 733 802 yen, 32 588 997 yen, and 33 444 192 yen, respectively, in the TAI group as compared with 32 256 095 yen in the SBC group, which suggest a reduced cost of 522 293 yen and additional costs of 332 902 yen and 1 188 097 yen, respectively, with TAI usage. The ICER of the TAI group when compared with that of the SBC group was dominant for the TAI procedure fee at 8100 yen. The ICER of the TAI group when compared with that of the SBC group was 417 994 yen/QALY and 1 491 781 yen/QALY for the TAI procedure fee at 13 050 yen and 18 000 yen, respectively.

### 3) Sensitivity Analysis

**Figure 2:**
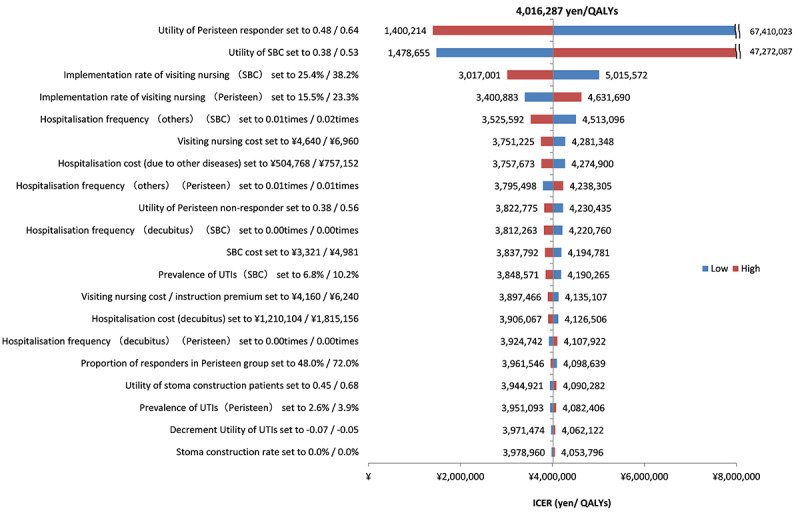
Tornado Diagram SBC: standard bowel care; UTI: urinary tract infections; QALYs: quality-adjusted life years; ICER: incremental cost-effectiveness ratio

**Figure 3:**
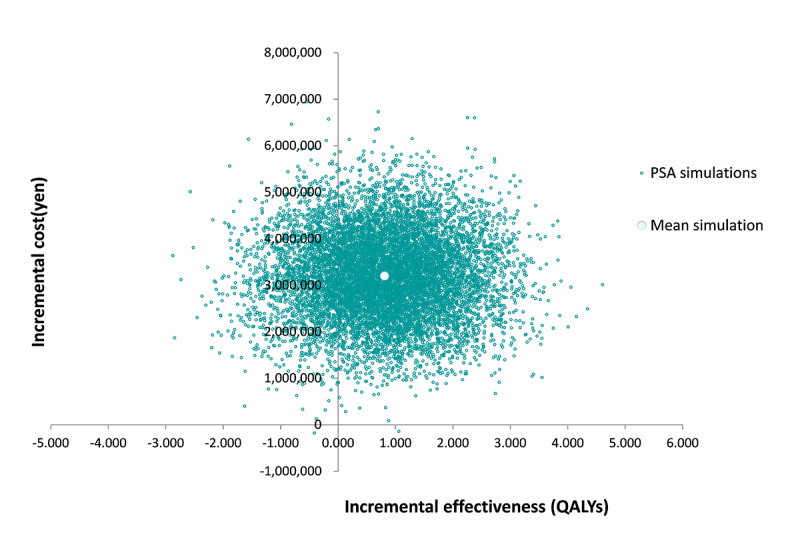
Cost-effectiveness Plane QALYs: quality-adjusted life years; ICER: incremental cost-effectiveness ratio

**Figure 4:**
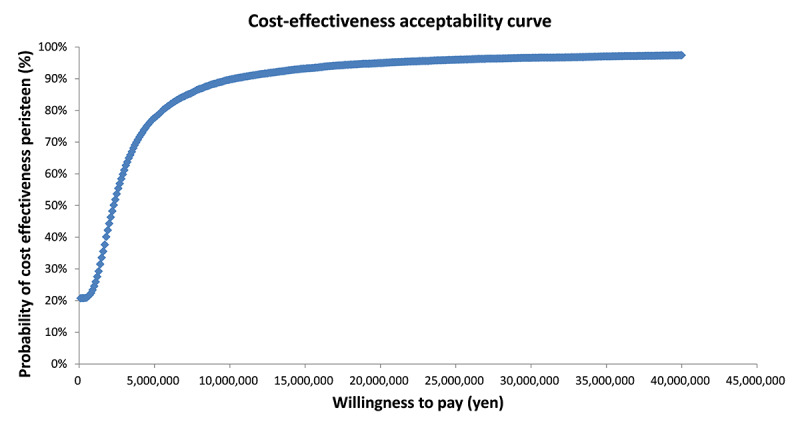
Cost-effectiveness Acceptability Curve

The results of the sensitivity analysis are shown in Figure 2, Figure 3, and Figure 4. The one-way sensitivity analysis for each parameter indicates that the utility weight for patients responding to TAI-treatment has the largest impact on the analysis outcome. However, using +/−20% of the base case setting as range for the utility weight for sensitivity analysis means that the worst-case scenario becomes rather unrealistic since it implies a similar or worse utility weight of TAI responders as that of SBC patients. Based on the probabilistic sensitivity analysis the cost-effectiveness acceptability curve shows that TAI can be considered as cost-effective in >50% of chance when the willingness to pay (WTP) of the decision maker was more than approximately 2 million yen.

## Discussion

In this study, a Markov model was used to analyze the cost-effectiveness of Peristeen usage for bowel management in SCI patients with NBD. The base case analysis reveals an overall ICER of 4 016 287 yen/QALY for patients using TAI as compared with those subjected to SBC.

In general, to determine cost-effectiveness, the ICER is expected to be lower than a predetermined threshold for the maximum societal WTP for a unit of additional health benefit. In Japan, however, there is currently no defined standard ICER threshold. In the UK, the National Institute for Health and Care Excellence (NICE) has communicated an ICER threshold ranging from £ 20 000–30 000/QALY (at a currency conversion rate of 1 £ = 143 yen, the range in Japan would be 2.86 million yen/QALY to 4.29 million yen/QALY).^24^ In the US, published reference values range from $20 000/QALY to $100 000/QALY (at a currency conversion rate of 1 $ = 120 yen, the range in Japan would be 2.4 million yen/QALY to 12 million yen/QALY).^25^ In addition, the World Health Organization (WHO) has proposed a method for calculating ICER thresholds by taking into account the gross domestic product (GDP) per capita of a country (GDP per capita in Japan: 3 850 000 yen).^26^ A product/service is usually considered “highly cost-effective” if the ICER threshold is lower than the GDP per capita, “cost-effective” if the ICER threshold is within the range of 1 to 3 times the GDP per capita (ranging from 3.85 million yen to 10.7 million yen), “not cost-effective” if the ICER threshold exceeds 3 times GDP per capita (more than 10.7 million yen).^28^ In Japan, investigations by Okusa *et al*. and Shiroiwa *et al*. reported that the maximum WTP per QALY gain^28^–^30^ to be 6.7 million yen and 5 million yen, respectively. The ICER thresholds in other countries and the reported WTP per QALY in Japan are listed in Table 9.

When comparing the ICERs obtained in this analysis with the WTP per QALY or ICER thresholds presented by Japanese and foreign research as well as authorities such as NICE, TAI is clearly a cost-effective treatment strategy in comparison with SBC in a Japanese setting. While the base case analysis gives an ICER just above the “highly-cost effective” GDP threshold of 3 850 000 yen suggested by the WHO, it falls well below the cost-effectiveness thresholds previously reported in Japan. In addition, when considering the overall societal perspective including productivity loss, all the analysis becomes “highly cost-effective,” and one scenario even found it to be cost saving, which means that TAI is both more effective and less costly than SBC.

A previously published cost-effectiveness analysis of TAI vs SBC from the United Kingdom^17^ was used as reference for our model. The study from the UK found that TAI reduced total cost and increased the expected QALYs in NBD patients. The present analysis is consistent with these results in terms of increase in expected QALYs, but not in terms of cost, where TAI proved to be costlier than SBC in Japan. One possible reason for the inconsistency could be that this analysis reflected a Japanese clinical setting which required adaptation of the basic model structure of the previous study.^17^ Another possible reason could be the difference in expenses reported by Emmanuel *et al*., in which the cost of Peristeen-TAI use was lower (approximately 850 dollars/cycle), whereas that of SBC was higher (approximately 1000 dollars/cycle), which is in contrast to the findings of this study.^17^ Moreover, in the original model from the United Kingdom, anal plug and diapers/pads were considered under SBC,^17^ whereas in Japan they are not covered by the insurance reimbursement system and were hence excluded from the analysis. With respect to the cost of Peristeen-TAI, adequate procedure reimbursement was found to be essential for the spread of TAI in Japan. However, in the model of Emmanuel *et al*., only the cost of the Peristeen TAI-device was considered,^17^ which results in the cost differences reported in this study.

This study has several limitations. The expected utility weight corresponding to each NBDS was estimated and used in the model. It would have been more appropriate to use utility data from Japanese patients using Peristeen-TAI. However, TAI-systems, such as Peristeen, have not yet been formally introduced in Japan, and the health benefits from TAI usage were therefore estimated using the mapping algorithm based on the Japanese patient survey.

Another possible limitation of this study is that the stoma creation rates have been calculated by regression analysis of the patient survey results and not on actual trial data. However, the sensitivity analysis showed that the influence of stoma incidence rate on the overall findings was negligible.

A final limitation concerns the general evidence base for the impact of TAI. Several studies have documented the distinct advantages of TAI in significantly reducing NBD symptoms in patients and improving QOL as compared with that achieved with SBC.^16^,^19^,^31^ This data has been generated in a variety of neurological diseases, but only one of the studies is a RCT and several of them contain only limited participants. These limitations in the available evidence on bowel management warrants further investigation on the effectiveness of TAI based on solid clinical trials including generic tools for measuring the impact on health-related QOL.

In conclusion, the cost-effectiveness of TAI for bowel management in SCI patients was analyzed using a Markov model. The results show that the ICER for TAI is clearly within the published thresholds in Japan as well as in other comparable countries. The main finding of this analysis is therefore that TAI is a cost-effective treatment strategy in Japan compared with SBC. Currently, evidence on the effectiveness of irrigation systems and that of TAI are mostly observational in nature. Therefore, further studies of TAI in clearly stratified Japanese NBD patients would be helpful to additionally describe the advantages of TAI. As described above, some of the parameters used in this study are based on non-Japanese sources, albeit that these are the validated sources used in previous publications of transanal irrigation. However, to increase validity it is desirable that data from Japanese people and sources should be used in the analysis. Therefore, when TAI is introduced in Japan and enough data has been collected, it is recommended that the current analysis be repeated.

## Figures and Tables

**Table 1:** Characteristics of Patients who Answered the Questionnaire and Included in the Analysis

Parameter	Value
N	217
Age, mean (SD)	51.46 (13.50)
Min	19
Max	87
Male, N (male %)	170 (78.3)
NBDS, mean (SD)	16.74 (5.72)
NBDS category, N (%)	-
NBDS: Minor (7–9)	23 (10.6%)
NBDS: Moderate (10–13)	44 (20.3%)
NBDS: Severe (Over 14)	150 (69.1%)

SD: standard difference; Min: minimum value; Max: maximum value; NBDS: neurogenic bowel dysfunction score

**Table 2:** Clinical Parameters

Parameter	Value		Reference
	SBC	Peristeen	
NBDS improvement effect	-	-	-
Responder	-	−6	Emmanuel data set data
Non-responder	-	+1	
Prevalence of UTIs/6 months	0.512 times	0.195 times	Patient survey, Christensen *et al*, 2009 (RR=0.381)^18^
Implementation rate of visiting nursing	31.80%	19.41%	Patient survey
Hospitalization rate	-	-	-
Hospitalization due to decubitus	0.31 times/patient	0.14 times/patient	Patient survey, Emmanuel data set (RR=0.44)
Hospitalization due to diseases other	than decubitus	1.73 times/patient	0.77 times/patient
Patient proportion using Peristeen	-		-
Responder	60%		Christensen *et al*, 2009^18^
Non-responder	40%		
Withdrawal rate of Peristeen/6 months	60%		Christensen *et al*, 2009^18^
Stoma construction rate/6 months	0.033%		Patient survey
Condition at stoma construction	-		-
Laparoscopic colostomy	57.5%		Medical specialist survey
Non-laparoscopic colostomy	42.5%		
Visiting nursing frequency/week	2 days		Assumed value
Treatment rate for UTIs	-		-
Hospitalization rate	8.6%		Medical specialist survey
Outpatient rate	81.1%		
Outpatient rate (no treatment)	10.3%		

SBC: standard bowel care; NBDS: neurologic bowel dysfunction score; UTI: urinary tract infection; RR: relative risk

**Table 3:** Utility Parameters

Parameter	Utility	Reference
Peristeen	-	-
Responder	0.533	Patient survey
Non-responder	0.470	
SBC	0.479	
Stoma	0.564	
Decrement utility value due to UTIs	−0.060	NICE, 201224
Decrement utility value in hospitalization	−0.010	Watt *et al*, 2012^32^

SBC: standard bowel care; UTI: urinary tract infection; NICE: National Institute for Health and Care Excellence.

**Table 4:** Cost Parameters

Parameter	Cost (yen)	Reference
Peristeen cost	-	-
Device cost/year	280 945	Peristeen system: 12 095 yen/set Peristeen Accessory Unit: 1426 yen/unit Annual use: Peristeen system 2 sets (24 190 yen) + Peristeen Accessory Unit 180 units (256 755 yen) ^*^As a device cost of Peristeen, the average of market prices in UK, Germany and France was used.
Procedure fee/year	216 000	The medical fee C106 home self-catheterization care instruction fee: 18 000 yen Postulate that it is calculated once a month
SBC cost/month	4151	Medical specialist survey
Stoma construction fee	-	-
laparoscopic colostomy	139 200	The medical fee K726-2 laparoscopic colostomy: 139 200 yen K726 Colostomy: 79 800 yen ^*^The weighted average value of the rate of laparoscopic colostomy and the rate of non-laparoscopic colostomy was used in the model. (113 955 yen)
non-laparoscopic colostomy	79 800	
Hospitalization cost at stoma construction	-	Medical specialist survey The weighted average value of the rate of laparoscopic colostomy and the rate of non-laparoscopic colostomy was used in the model. (239 020 yen)
laparoscopic colostomy	236 311	
non-laparoscopic colostomy	242 686	
Stoma management cost/month	-	Medical specialist survey
1st year	4131	
From 2nd year	1621	
Hospitalization cost for UTIs/month	163 113	Medical specialist survey The weighted average value of hospitalization cost for UTIs UTI, medical care expenditure of UTIs outpatients and Medical care expenditure of UTIs outpatients was used in the model. (26 507 yen)
Medical care expenditure of UTIs outpatients/month	14 126	
Medical care expenditure of UTIs outpatients(no treatment)/month	10 293	
Visiting nursing cost/day	5800	The medical fee C005 Visiting nursing/instruction fee (per day) (for 3 weeks): 5800 yen
	5200	The medical fee Long-hours visiting nursing/instruction premium (only once a week): 5200 yen
Hospitalization cost	-	-
Due to decubitus	1 512 630	MDV database
Due to other diseases	630 960	

UK: the United Kingdom; SBC: standard bowel care; UTI: urinary tract infection; MDV: Medical Data Vision Inc.

**Table 5:** Characteristics of Patients Included in the MDV Data Analysis

	n, mean	%, SD
N	3553	-
Sex, n, %		
Male	2623	73.82%
Female	930	26.18%
Age, mean, SD	65.87	14.57
Comorbidities, n, %		
Respiratory disease	1352	38.05%
Cardiovascular disease	2214	62.31%
Infectious diseases	1104	31.07%
Immune deficiencies	25	0.70%
Diabetes	1128	31.75%
Diseases of the genitourinary system	1748	49.20%
Concomitant drugs, n, %		
Glycerin enema	944	26.57%
Colonel tablets	15	0.42%
Teleminsoft suppositories	258	7.26%
Bio-three	64	1.80%
Pursennid tablets	1196	33.66%
Laxoberon solution	515	14.49%
Loperamide hydrochloride	57	1.60%
Magnesium oxide	1054	29.67%
New lecicarbon supp.	361	10.16%
Major middle-strengthening decoction	204	5.74%
Cravit fine granule	3	0.08%
Cravit tablets	404	11.37%
Kefral	38	1.07%
Bactramin combination tablet	95	2.67%
Firstcin intravenous	25	0.70%
Flomox	273	7.68%
Bladderon tablets	4	0.11%
Minomycin tablets	74	2.08%
Cravit intravenous drip infusion	38	1.07%
Zosyn	187	5.26%
Pentcillin	76	2.14%
Fosmicin-s for injection	26	0.73%
Unasyn-s for intravenous use	278	7.82%

MDV: Medical Data Vision Inc.; SD: standard difference

**Table 6:** Productivity Loss Parameters

Parameter	Value	Reference
Employed rate	48.8%	Patient survey
Absenteeism	-	
NBDS : Minor (7–9)	0.00%	
NBDS : Moderate (10–13)	2.21%	
NBDS : Severe (Over 14)	7.01%	
Presenteeism	-	
NBDS : Minor (7–9)	17.00%	
NBDS : Moderate (10–13)	36.36%	
NBDS : Severe (Over 14)	39.71%	
Scheduled wage/month	-	Basic Survey on Wage Structure in 2015 Annual Report on Government Measures for Persons with Disabilities in 2012 ^*^Scheduled wage per month was adjusted with the proportion of average wage per month of the disabled and regular workers reported in Annual Report on Government Measures for Persons with Disabilities to scheduled wage reported in Basic Survey on Wage Structure.
Age 50~54	364 163 yen	
Age 55~59	350 789 yen	
Age 60~64	262 659 yen	
Age 65~69	244 283 yen	
Age 70~	248 227 yen	

NBDS: neurogenic bowel dysfunction score

**Table 7:** Base Case Analysis Results

	Cost (yen)	Incremental cost (yen)	QALYs	Incremental QALYs	ICER (cost/yen)
**SBC**	12 532 111	-	11.00	-	-
**Peristeen**	15 730 798	3 198 687	11.80	0.80	4 016 287

SBC: standard bowel care; QALYs: quality-adjusted life years; ICER: incremental cost-effectiveness ratio

**Table 8:** Scenario Analysis

	Cost (yen)	Incremental cost (yen)	QALYs	Incremental QALYs	ICER (cost/yen)
Absenteeism					
SBC	15 482 680	-	11.00	-	-
Peristeen	17 474 384	1 991 704	11.80	0.80	2 500 793
Absenteeism+Presenteeism					
SBC	32 256 095	-	11.00	-	-
Peristeen	33 444 192	1 188 097	11.80	0.80	1 491 781

SBC: standard bowel care; QALYs: quality-adjusted life years; ICER: incremental cost-effectiveness ratio

**Table 9:** ICER Thresholds in other Countries and the Reported Willingness to Pay per QALY in Japan

	Value (original currency)	Value (converted to yen)*
**ICER thresholds in other countries**	**-**	**-**
NICE	£20 000 – 30 000	2.86 – 4.29 million yen
US	$20 000 – 100 000	2.2 – 11 million yen
WHO	Per capita GDP - 3 times per capita GDP	3.85–10.7 million yen
**Reported WTP per QALY in Japan**	**-**	**-**
Ohkusa *et al*, 2006	6.7 million yen	6.7 million yen
Shiroiwa *et al*, 2010	5 million yen	5 million yen

ICER: incremental cost-effectiveness ratio; QALY: quality-adjusted life year; NICE: National Institute for Health and Care Excellence; US: the United States; WHO: World Health Organization; GDP: gross domestic product.

* £1 = 143 yen, $1 = 120 yen.
